# Nicotinamide Inhibits Vasculogenic Mimicry, an Alternative Vascularization Pathway Observed in Highly Aggressive Melanoma

**DOI:** 10.1371/journal.pone.0057160

**Published:** 2013-02-25

**Authors:** Orit Itzhaki, Eyal Greenberg, Bruria Shalmon, Adva Kubi, Avraham J. Treves, Ronnie Shapira-Frommer, Camilla Avivi, Rona Ortenberg, Eytan Ben-Ami, Jacob Schachter, Michal J. Besser, Gal Markel

**Affiliations:** 1 Ella Institute of Melanoma, Sheba Medical Center, Ramat-Gan, Israel; 2 Department of Clinical Microbiology and Immunology, Sackler Faculty of Medicine, Tel Aviv University, Tel Aviv, Israel; 3 Cancer Research Center, Sheba Medical Center, Ramat-Gan, Israel; 4 Department of Pathology, Sheba Medical Center, Ramat-Gan, Israel; 5 Talpiot Medical Leadership Program, Sheba Medical Center, Ramat-Gan, Israel; University of Porto, Portugal

## Abstract

Vasculogenic mimicry (VM) describes functional vascular channels composed only of tumor cells and its presence predicts poor prognosis in melanoma patients. Inhibition of this alternative vascularization pathway might be of clinical importance, especially as several anti-angiogenic therapies targeting endothelial cells are largely ineffective in melanoma. We show the presence of VM structures histologically in a series of human melanoma lesions and demonstrate that cell cultures derived from these lesions form tubes in 3D cultures *ex vivo*. We tested the ability of nicotinamide, the amide form of vitamin B3 (niacin), which acts as an epigenetic gene regulator through unique cellular pathways, to modify VM. Nicotinamide effectively inhibited the formation of VM structures and destroyed already formed ones, in a dose-dependent manner. Remarkably, VM formation capacity remained suppressed even one month after the complete withdrawal of Nicotimamid. The inhibitory effect of nicotinamide on VM formation could be at least partially explained by a nicotinamide-driven downregulation of vascular endothelial cadherin (VE-Cadherin), which is known to have a central role in VM. Further major changes in the expression profile of hundreds of genes, most of them clustered in biologically-relevant clusters, were observed. In addition, nicotinamide significantly inhibited melanoma cell proliferation, but had an opposite effect on their invasion capacity. Cell cycle analysis indicated moderate changes in apoptotic indices. Therefore, nicotinamide could be further used to unravel new biological mechanisms that drive VM and tumor progression. Targeting VM, especially in combination with anti-angiogenic strategies, is expected to be synergistic and might yield substantial anti neoplastic effects in a variety of malignancies.

## Introduction

Melanoma, an aggressive malignancy arising from neural crest melanocytes, is the most lethal form of skin cancer worldwide. Melanoma is considered as the most fatal malignancy of young adults and its incidence has increased dramatically over the last 50 years [1], [2]. Despite significant breakthroughs in understanding the pathobiology of melanoma, which resulted in new therapies [3], [4], disseminated melanoma is still a considerable clinical problem due to the complexity of targeting the elusive metastatic phenotype [5].

The plastic notion of melanoma is characterized by the concurrent expression of genes from a variety of different cell types, including stem cells, concomitantly with reduced melanoma associate gene expression [6], [7]. In particular, highly aggressive melanoma cells, in contrast to poorly aggressive ones, display substantial plasticity, exemplified by the formation of tube-like structures termed “Vasculogenic Mimicry” (VM) [8]. These structures are comprised exclusively of tumor cells but not of endothelial cells, and conduct blood cells and fluids.

VM describes the ability of tumor cells to express endothelium- and epithelium-associated genes and to form extracellular matrix (ECM)–rich tubular networks in three-dimensional cultures [8] that “mimic” the pattern of embryonic vasculogenic networks independently of angiogenesis [9], [10]. Histologically, VM emerges as multiple, laminin-rich networks surrounding clusters of tumor cells, which can be stained with periodic acid-Schiff (PAS) [11]. The formation of VM channels is not an angiogenic event as it does not arise from pre-existing vessels, and despite the fact that VM channels develop *de novo* – a feature shared with vasculogenesis – they are clearly not blood vessels [12]. VM or a PAS-positive pattern are also associated with tumor aggressiveness, poor clinical outcome, and high risk of recurrence in patients with melanoma [8], [13], [14] and other malignances [15], [16], [17], [18], [19].

The fact that VM is an angiogenesis-independent mechanism, could explain why anti-angiogenic therapies have clinically failed in melanoma (reviewed in [20]), despite being macroscopically a highly “vascular” tumor. Indeed, traditional anti-angiogenic drugs, such as endostatine, have been ineffective at inhibiting VM [21], [22]. Since VM is an alternative pathway for tumors to guarantee their blood supply, it is necessary to find potential therapeutic approaches that could target this alternative vascular pathway.

VE-cadherin, ephrins, focal adhesion kinase, phosphatidyl inositol-3-kinase, Galectin-3, and Nodal (reviewed in [23], [24] and [25]) have been identified as molecules playing a central role in VM formation and signaling. Currently, anti VM therapies are proposed by several groups. Most of them aim to either remodel the ECM and tumor microenvironment, to block biochemical and molecular signaling pathways of VM (Reviewed in [26]) or to inhibit tumor cell plasticity. Targeting Nodal, an embryonic morphogen that contributes to metastatic melanoma cell plasticity and tumorigenesis, addresses this approach [27]. Nevertheless, VM formation could qualify as a trans-differentiation process of a subpopulation of melanoma cell with epigenetic regulation [28].

Nicotinamide (NA) is the amide form of vitamin B3 (niacin), which is a component of the coenzymes nicotinamide adenine dinucleotide (NAD) and its phosphate form, NADP. NA directly impacts normal physiology due to its role in the cellular energy metabolism. NA also influences oxidative stress and modulates multiple pathways related to both cellular survival and death [29]. Additionally, this agent has an anti-inflammatory Th1 to Th2 switching effect [30] and the ability to block pro- inflammatory signal transduction pathways and mediators *in vitro* [29].

NA acts as an epigenetic gene regulator through unique cellular pathways via direct inhibition of four classes of enzymes, including the histone deacytylases (sirtuin) main member SIRT1 [29], [31]. In cancer increased SIRT1 expression and function is followed, among other effects, by a decrease in the p53 active form which leads to genomic instability and resistance to apoptosis [32]. Particular in skin cancer, NA was shown to protect against UV-induced immunosuppression in animal models as well as in humans [33], [34] and significantly reduced the incidence of UV-induced skin tumor in mice [33].

Current research suggests that NA, or vitamin B3, may play a key role in cancer prevention via its activation in cellular repair [35]. NA was extensively studied over the years for its radio-sensitizing properties [36], [37], and a recent report indicated an increased efficacy of radiotherapy in locally advanced bladder carcinoma by NA [38]. In conclusion, NA has broad activities on many cell types, including regulation of cell adhesion, polarity, migration, proliferation and differentiation, and most importantly, has a de-differentiating function on differentiated cells [29], [39]. Thus, we hypothesized that NA could qualify as a suitable agent for VM inhibition.

Here we demonstrate the existence of the VM phenomenon in cutaneous melanoma sections and their parallel low-passage primary melanoma cultures established in our laboratory. We further show the ability of NA to abrogate VM formation at the molecular and functional levels, as well as to alter other features of melanoma cells such as proliferation and invasion.

## Results

### Morphological Characteristics and Quantification of VM

**Figure 1:**
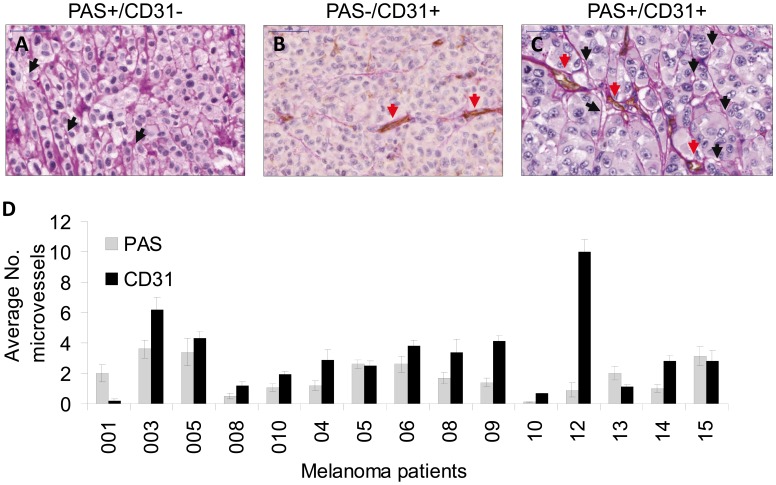
Characterization of vessel with PAS-CD31 dual staining (endothelial or vessel like channels – VM) in paraffin sections of melanoma patients. (A) PAS-positive VM channels with no endothelial marker CD31 staining, black arrows indicate VM channels; (B) PAS-negative patterns and CD31-positive staining; red arrows indicate endothelial channels; (C) PAS-positive and CD31-positive channels; (D) a summary of microscopic vessels observed. Microscopic evaluation was done blindly by two pathologists. The data represents average values as seen in 10 high power fields per sample.

Histologically, VM appears as multiple, laminin-rich PAS positive networks and surround clusters of tumor cells [11]. In order to verify the identification of VM structures *in situ*, we combined CD31 staining, to identify endothelial cells, and PAS staining, to determine the basement membranes of micro-vessels. Any structure containing CD31-positive immunoreactivity was defined as a blood vessel, while VM structures were strictly defined as CD31-negative PAS-positive structures (Figure 1, A–C). CD31-PAS double staining was performed on 15 tissue samples derived from melanoma patients (Table 1 and Table S1). The potential pathophysiological relevance of VM channels was evaluated by comparing their abundance to that of CD31-positive blood vessel in each of the melanoma specimens. Notably, VM structures were detected in 14 of the 15 melanoma specimens (Figure 1D) and comprised a remarkable proportion of 40% of the total number of blood vessels, on average. These observations emphasize the potential importance of vasculogenic mimicry for melanoma development. There was no apparent correlation with any of the clinical parameters.

**Table 1:** Vasculogenic mimicry characteristics.

	PAS positive (%)paraffin section*	Tube formation(Matrigel)		VE-Cadherin (%)**
**Primary melanoma**				
001	91	−	14±3.0	
003	37	++	38±7.0	
005	44	++	44±7.0	
008	29	++	48±3.0	
010	78	+++	43±3.0	
04	29	−	19±5.0	
05	51	++	27±3.0	
06	41	−	58±9.0	
08	33	+	15±0.0	
09	25	++	19±6.0	
10	13	+	50±24	
12	5	+++	17±0.0	
13	65	+++	38±7.0	
14	26	+++	28±12	
15	53	+	31±4.0	
**Established cell lines**				
HAG	nd	+++	80±2.7	
PAG	nd	−	0.4±0.2	
**Primary cultures**				
HUVEC	nd	+++	75±9.0	
NHEM	nd	−	0.3±0.05	

nd = not determined.

* CD31-PAS double-staining calculate as N_VM structures_/(N_VM structures_+N_CD31-positive structures_) ×100.

** Percentage of VE-cadherin expression was determined by FACS analysis.

### Capillary-like Formation in Three-dimensional Cultures of Cell Lines and Primary Melanoma Cell Cultures

**Figure 2:**
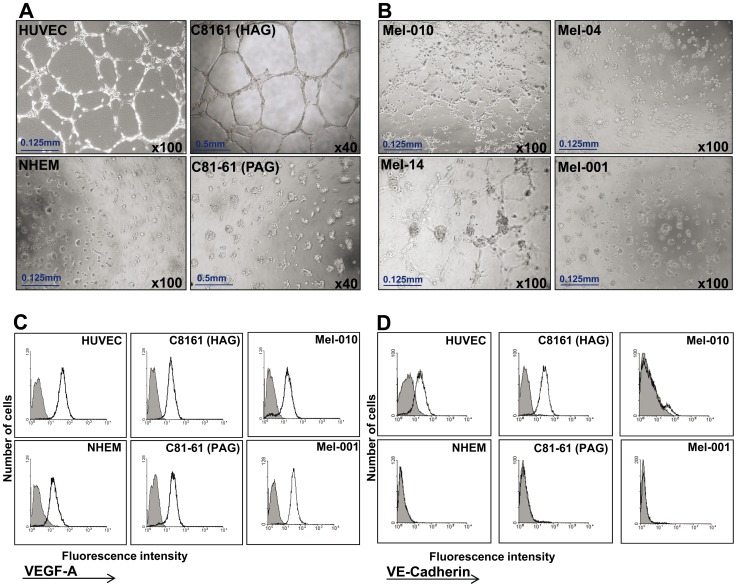
Tube formation activity in vitro by low-passage primary melanoma cultures. (A) Vessel-like networks in three-dimensional culture of cell lines: C8161 (HAG) and HUVEC (positive controls) and C81-61 (PAG) and NHEM (negative controls). Original magnification as indicated in images; (B) Vessel-like networks in three-dimensional culture of primary low-passage melanoma cultures: Mel-010 and Mel-14 (positive) and Mel-04 and Mel-001 (negative); (C) intracellular VEGF-A expression by indicated cells. Shaded histograms denote isotype control antibody only and empty histograms denote VEGF-A staining; (D) percentage of VE-cadherin positive cells as determined by flow cytometry. The data represents one of three experiments with equal results.

It was suggested before that melanoma cells capable of forming VM structures are characterized by an endothelial phenotype, which enables them to form tubular networks when grown in 3D culture *in vitro* [8]. Indeed, tube formation activity was previously demonstrated with endothelial HUVEC cells and the highly aggressive (HAG) C8161 melanoma cell line, which is the commonly studied human cutaneous melanoma model for vasculogenic mimicry [8], [22], [40], [41]. In contrast, the poorly aggressive (PAG) C81-61 melanoma cell line and normal human epidermal melanocytes (NHEM) did not form any tubes or networks (Figure 2A). Low-passage primary melanoma cultures (Table 1), derived from metastatic melanoma patients (Table S1) were tested for tube formation in matrigel. Several cultures exhibited a tube formation activity, while other cultures failed (Table 1, exemplar cultures shown in Figure 2B). There was no evidence for correlation between the VM capacity *in situ* and tube formation activity *in vitro (p = 0.698).*

VEGF-A is a known driver for tumor vasculogenesis [42], but a recent report suggested it has a role in VM as well [43]. Intracellular staining for VEGF-A content in HAG, PAG, HUVEC, NHEM and low-passage primary cultures Mel010 and Mel001 demonstrated its presence in all tested cells (Figure 2C). All cell cultures similarly expressed VEGF-A (MFI ranged from 10 to 35), except for HUVEC cells, which expressed a higher content of VEGF-A (MFI = 51) (Figure 2B). Therefore, the differential VM activity of melanoma cells cannot be entirely explained by different expression levels of VEGF-A (Figure 2C).

Based on the HAG and PAG model, it was previously reported that tube formation activity of tumor cells is associated with expression of VE-cadherin [44]. Indeed, almost all HAG cells expressed VE-cadherin, while essentially none of the PAG cells were VE-cadherin positive (Figure 2C and Table 1). In most of the primary melanoma cultures some VE-cadherin positive cells were identified (Figure 2D), but their frequency did not correlate to *in vitro* tube formation levels (p = 0.867) (Table 1). In addition, VE-cadherin expression *in vitro* did not correlate with VM capacity *in situ (p = 0.998).* In established cell lines on the other hand, VE-cadherin expression directly correlated with the ability to form VM channels on 3D matigel (Table 1).

### Nicotinamide Induces a Robust Alteration in Gene Expression Profile Related to Vasculogenic Mimicry

**Figure 3:**
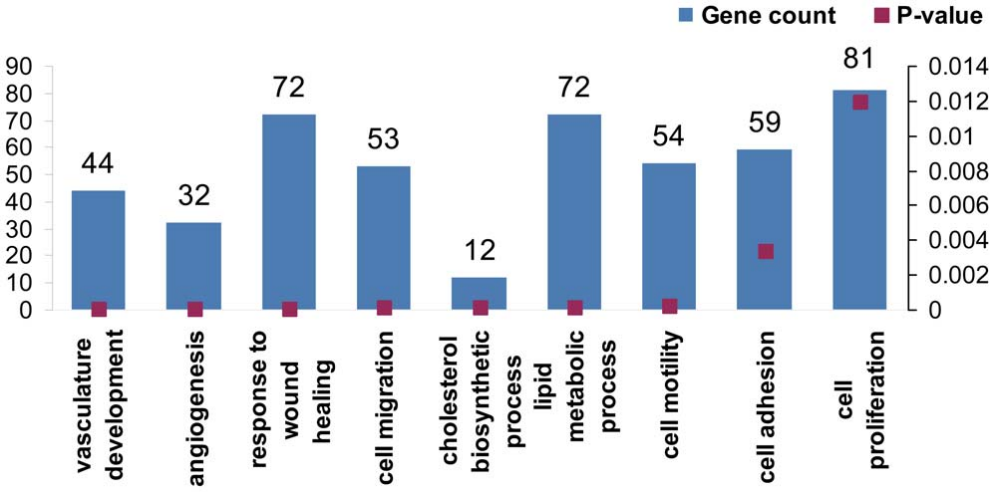
Microarray analysis of NA-treated melanoma cells. Figure shows the top altered genetic clusters according to Toppgene algorithm.

Nicotinamide (NA) acts as an epigenetic gene regulator with a de-differentiating function on differentiated cells. HAG cells treated with 20 mM of NA for 3 days were subjected to whole genome expression microarray and compared with untreated control cells. A substantial number (1122) of significantly altered genes (>2-fold or <0.5-fold) was found. Remarkably, four of the nine most prominent gene clusters affected seem to have a close relation to vasculogenic mimicry: vasculature development, angiogenesis, cell migration and cell motility (Figure 3). More specifically, VE-cadherin (CDH5) was downregulated by 6.67 fold compared to control, as well as other key molecules such as VEGF-A, MMP2, TGF-b1 and SIRT5 (Table S2). These results strongly imply that NA has the potential to exogenously affect VM activity of melanoma cells in a consistent manner.

### NA Abrogates VM Activity of Melanoma Cells

**Figure 4:**
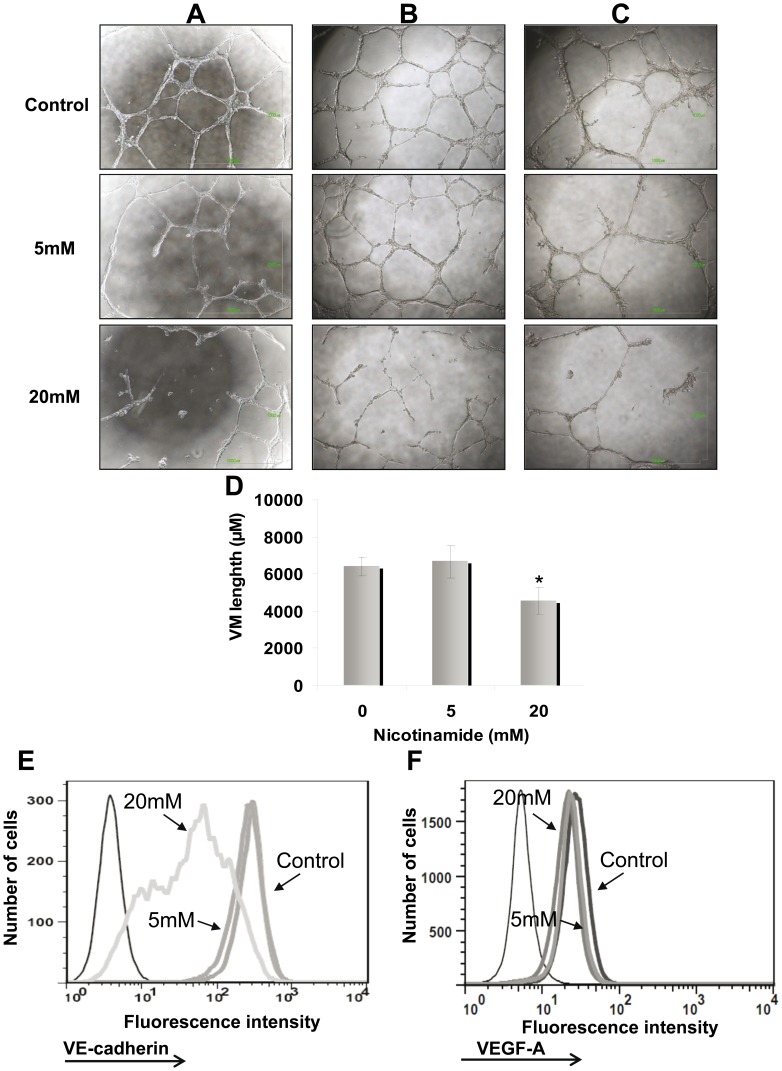
Nicotinamide (NA) abrogates vasculogenic mimicry. (A) NA was added 24 h after capillary-like structures were developed (destruction of VM formation). NA was added in concentrations of 5 mM and 20 mM as indicated for additional 24 h. Afterwards, microphotographs were captured; (B) HAG was cultured in the presence of NA (same concentrations as A) for 1 month (prevention of VM formation). Subsequently, the treated cells were cultured on Matrigel to enable VM formation. The images were captured after 24 h; (C). Prolonged inhibition of VM formation by NA treatment. The cells were incubated with NA for 72 h, re-plated without NA for 1 month, followed by VM testing. Microphotographs were capture 24 h after plating. Each picture (A–C) was derived from one representative experiment out of three performed. Each experiment was performed in duplicates; (D) Tube formation was quantified using the ImageJ analyze skeleton PlugIn. Figure showed the average length calculated for each sample out of all image captured in all three experiments performed. Statistical significant was tested with 2-tailed paired *t-test*. (E) Effect of NA on VE-cadherin expression in the HAG cells cultured for 1 month in the presence of NA. (F) Effect of NA on VEGF-A expression in the HAG cells cultured for 1 month in the presence of NA. Black thin line histogram represents cells stained with isotype control; other histograms represent cells treated either with vehicle or various NA concentrations, as indicated in the figure. Figure shows representative experiments out of three performed.

The effect of NA on VM activity was tested in two main setups: destruction of existing VM structures and prevention of tube formation. In the first experiments, HAG cells were seeded on matrigel and cultured for 24 h to allow development of VM structures. Then, NA was added onto the 3D culture in a final concentration of 5 mM and 20 mM. Vehicle-only served as negative control. A prominent dose-dependent destruction of pre-formed VM structures was observed (Figure 4A). In the second setup, HAG cells were cultivated for one month in the presence of 5 mM or 20 mM of NA, or vehicle-only, and tested for VM activity (without NA in the assay). Again, a prominent dose-dependent inhibition of VM activity was evident (Figure 4B). Finally, we tested the durability of the VM inhibitory effect by NA. HAG cells were exposed to the same NA concentrations as above for 72 hours, washed and further cultivated in NA-free medium. Strikingly, the inhibitory effect on VM formation was maintained in a dose-dependent manner even one month after NA withdrawal (Figure 4C). Viability was equally high in all cell cultures. Quantification of total tube length was performed using ImageJ. The qualitative assessment of micrographic captures (Figure 4 A–C) concurred with the quantitative total length analysis (Figure 4D). In agreement with the downregulation of VE-cadherin in the microarray and the VM inhibitory effects of NA, a dose-dependent downregulation of VE-cadherin was observed at the protein level (Figure 4E). In contrast, VEGF expression was not affected by NA treatment (Figure 4F).

It can be concluded that downregulation of VE-cadherin by NA at least partially explains the inability of melanoma cells to form vasculogenic-like networks in the presence of NA.

### Effect of NA on Proliferation, Invasion and Cell Cycle Profile of Melanoma Cells

**Figure 5:**
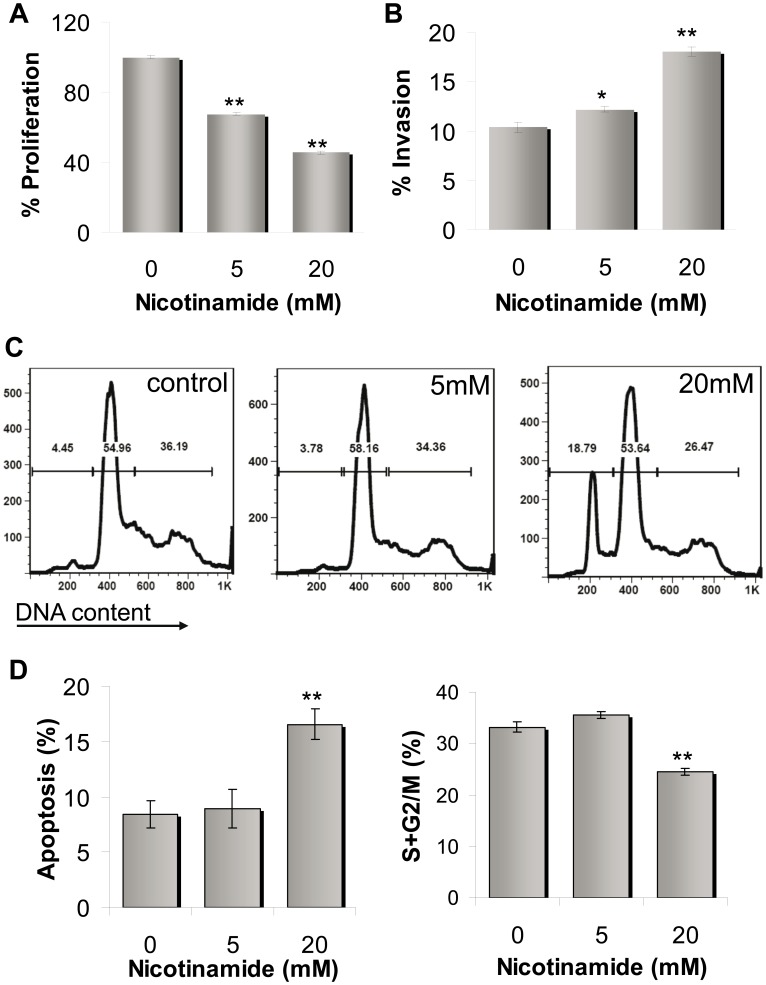
Effect of NA on proliferation, invasion and cell cycle profile of melanoma cells. (A) Net proliferation of HAG cells was quantified with standardized XTT test. The number of cells was determined 72 h after seeding. The number of the vehicle-treated control cells was determined as 100%. Figure shows a representative experiment out of four performed; (B) Invasion capacity of HAG cells was quantified by 24 h-matrigel invasion assay, with correction to proliferation. The invasion rate of the vehicle-treated control cells was determined as 100%. Figure shows a representative experiment out of three performed; (C) DNA flow cytometry of HAG cells in response to NA compared with vehicle-treated control cells-representative experiment; (D) Quantitative evaluation of the S+G2M subpopulation and the apoptotic cell fraction, measured as a subdiploid peak. Statistically differences compared with control. * indicate P<0.05, ** indicate P<0.01 (2-tailed t-test).

The effect of NA on the phenotype of melanoma cells, including proliferation, cell cycle profile and invasion, was tested. As shown in Figure 5A, a pronounced and statistically significant inhibition of net proliferation of HAG melanoma cells treated with NA was evident in a dose-dependent manner, as compared to untreated cells. These observations were confirmed by cell cycle profiling, which revealed moderate changes in apoptotic indices and percentage of cells in the S+G2M phase in NA-treated cells (Figure 5C and D). Interestingly, NA treatment enhanced the invasion activity of HAG cells, again in a dose-dependent manner (Figure 5B).

## Discussion

VM formed by highly aggressive melanoma cells is a novel form of tumor microcirculation pattern, which differs from classically described endothelium-dependent angiogenesis. Previous studies demonstrated the prognostic value of VM in primary melanomas, using metastatic recurrence as an endpoint [8], [13], [14]. It should be emphasized that all of specimens in this study were already derived from metastases, and indeed VM was abundantly identified in almost all of the specimens (Figure 1, Table S1). There were no clinical associations with additional parameters such as age, gender, site of metastasis, size of tumor etc. It is therefore hypothesized that VM enables resistance to conventional anti-angiogenic medicaments. Taking into consideration that VM is an example of tumor cell plasticity displaying highly dedifferentiated phenotype, targeting this phenomenon is a complex task. It has been demonstrate that several drugs could inhibit VM (reviewed in [26]). In this study, we found that NA could inhibit VM formation of the highly aggressive (HAG) melanoma cell line C8161 *in vitro*. NA also reduced proliferation and increased invasion capacity, as well as cause apoptosis to a certain degree. It is possible that these effects could contribute indirectly to the inhibition of VM. Notably, it was previously reported that VM is associated with invasion [28], which was increased here by NA, thus arguing against a significant indirect mechanism. In addition, VM was still inhibited even one month after washout of NA (Figure 4). Defining the molecular mechanisms underlying VM will further enable development of specific anti-VM strategies.

It was suggested that vascular channels in general, including VM channels, play a role not only in supplying oxygen and nutrients required for tumor growth, but also in enhancing tumor metastasis [7], [45]. We found in a series of clinical metastatic melanoma specimens both CD31-positive endothelial channels and CD31-negative PAS-positive VM channels. Importantly, the proportion of VM was, on average, similar to that of endothelial blood vessels, supporting a significant role for VM. In addition, we observed the unique architectures that were described by Folberg et al [46] based on PAS staining, which are: straight channels, parallel straight pattern, parallel straight pattern with cross link, arcs (not closed), arcs with branching, closed loops, and networks.

Most of the literature data on VM in human cutaneous melanoma emerges from investigations on the model of C8161 (HAG) and C81-61 (PAG) isogenic, homogenous, cell lines. We were able to demonstrate tube formation activity of low-passage primary cultures of metastatic melanoma however it was not correlative to the percentage of VM channels observed in the paraffin embedded biopsies. It was previously reported that VM activity is associated with the expression of VE-cadherin, based on studies with HAG (VE-cadherin-High) and PAG (VE-cadherin -Negative) cells [44], jointly with the tyrosine kinase receptor EphA2, which was proposed as initiator of the VM signaling cascade [40], [44], [47]. An association between the expression of VE-cadherin and VM activity *in vitro* was mainly evident among established cell lines, and less among low-passage primary cultures (Table 1). The latter might be explained by the phenotypic heterogeneity and sensitivity to environmental manipulation of low-passage primary melanoma cultures [48]. While *in vivo* a small proportion of VE-cadherin positive cells might suffice for generation of capillary-like structures, the *in vitro* conditions are probably more restrictive. This might also explain why there was no correlation between VM *in situ* and tube formation activity *in vitro*. Therefore, mechanistic VM studies *in vitro* are limited to selected cell lines with an extreme phenotype, such as HAG, evident by the bright VE-cadherin expression. Nevertheless, targeting VM is still attractive. With the challenge of finding new drugs which could inhibit VM, the soybeans isoflavone Genistein [49] was found to be able to inhibit VM formation of uveal melanoma through down-regulation of VE-cadherin *in vitro* [50]. In the present study, we show that treatment with NA downregulates VE-cadherin expression to inhibit VM activity. It could be proposed that NA abrogate channel-like structures directly by downregulation the most essential molecule for VM network formation. Indeed, VE-cadherin was downregulated significantly in both RNA (Table S2) and protein (Figure 4E) levels, whereas VEGF-A was downregulated at the RNA level (Table S2), but not in the protein level (Figure 4F).

Hypoxia was found to encourage tube formation *in vitro* and expression of genes associated with VM [51]. Consequently, VM is mostly resistant to anti-angiogenic therapy [21], [22], and is therefore probably involved metastasis through VM endorsement [52]. NA was found to act by decreasing perfusion-limited tumor hypoxia through prevention of intermittent vascular shut-down [36], [53]. Another possible mechanism of action *in vivo* for NA could be inhibition of VM formation through prevention of local hypoxia. Indeed, the VE-cadherin gene contains genomic binding sites for HIF-1α and HIF-2α transcription factors that are stabilized during condition of hypoxia [54]. It was proposed that HIF-2α expression in aggressive tumor cells is associated with de-differentiation towards the endothelial lineage by transcriptional induction of VE-cadherin [25].

The ability of plastic tumor cells to present an endothelial phenotype is probably due to the reactivation of embryonic signaling cascades causing aggressive tumor cell to dedifferentiate [55]. In addition, melanoma cells appear to reversibly generate stem-like cells through “phenotype switching”. By this process, melanoma cells can switch between migratory, stem-like state and proliferative state in response to changes in microenvironmental conditions [56]. In the present study we observed that NA inhibited proliferation and enhanced invasiveness of HAG cells *in vitro* (Figure 5). It could imply that NA has a potential role in phenotype switching, but this must be thoroughly investigated in primary cultures in future studies (Figure 5). In a recent review, Girouard and Murphy [57] proposed that the undifferentiated, primitive, embryonic like stem cells profile of aggressive melanoma cells engage in VM, implying that melanoma stem cell may give rise to the patterned networks that characterize VM. Monzani et al [58] showed that a stem cell population that potentially increases tumor progression, is found in melanoma biopsies. Thus, it is suggested that cancer stem cell (CSC) subpopulation inside the tumor are capable of organizing VM networks, depending on the environmental condition. All of these contribute to the failure of current therapeutic regimens by masking malignant target populations.

It has been observed in a mouse model that invasive phenotype cells escape melanoma allograft in large numbers [59]. Hoek and Goding [56] suggested that cells with invasive phenotype possibly submitted to senescence or apoptosis and only a fraction survive to switch back to the proliferative state. Alternatively, they proposed that the switch from invasive to proliferative is very low, which may lead to dormancy over long periods. The complexity of the metastatic phenotype, including heterogeneity and phenotype-switching, with plasticity close to that of embryonic cells, must be taken into account when designing new therapies. NA addresses the need for designing such strategies combined with other modalities, including the molecular based approach aimed to suppress the aggressive melanoma phenotype in individual patients [60]. It was shown that NA had an effect of the differentiation of leukemia cells through its histone deacetylase inhibitory activity [61]. Recent studies in experimental human tumors showed antiproliferative proapoptotic activities by NA [62] and pronounced inhibition of growth and progression [63]. It is highly likely that the broad alterations in gene expression we observed in melanoma following exposure to NA and the subsequent functional effects are due to the epigenetic regulation exerted by NA, e.g. by histone deacetylase inhibition. Thus, NA could be quilted as VM-targeted strategy in addition to its wide range of biological activities and its different effects mediated by different concentration. The combination of anti-VM agents like NA with other therapeutic strategies is expected to yield the best results.

In conclusion, this study shows that NA could successfully inhibit the VM formation of C8161 human cutaneous melanoma. One mechanism in which NA inhibits VM is associated with downregulation of VE-cadherin. Moreover, NA inhibits proliferation and increases invasiveness and apoptosis in melanoma cells. This study may provide preliminary evidence for future and wider research to elucidate the mechanisms underlies VM inhibition by NA and its mode of action.

## Materials and Methods

### Patients

Melanoma lesions were surgically removed from 15 patients participating in a clinical study at the Ella Institute, Sheba Medical Center following approval of the local ethics committee of Sheba Medical Center, Tel Hashomer, Israel (Israeli Ministry of Health approval No. 3518/2004). All patients signed an informed consent form. Clinical characteristics of the melanoma patients are described in Table S1.

### Cells

Primary melanoma cultures were established from the surgically removed melanoma lesions as previously described [64], [65]. The tumor cultures were grown in RPMI (Lonza, Verviers Sprl, Belgium) containing 10% fetal bovine serum (FBS) (Gibco Minneapolis MN, USA), 25 mmol/l HEPES pH 7.2 (Lonza), 100 U/ml penicillin (Lonza), 100 mg/ml streptomycin (Lonza) and 1 mM sodium pyruvate (Lonza). Normal human epidermal melanocyte (NHEM) and human umbilical vein endothelial cells (HUVEC) was purchased from Promo Cell (Heidelberg, Germany). The cells were plated in endothelial growth medium or melanocyte growth medium, respectively, supplemented with growth factor mixture (Prom Cell) according to the manufacturer’s recommendation. The human cutaneous melanoma cell lines C8161 (highly aggressive-HAG) and C81-61 (poorly aggressive-PAG) were kindly provided by Dr. Marry Hendrix (Children’s Memorial research Center, Chicago, IL, USA) [66]. HAG cells were grown in RPMI as described above, while PAG cells were plated in Ham’s F10 medium (Biological Industries, Bet Haemek, Israel) supplemented with 15% FBS, 100 U/ml penicillin, 100 mg/ml streptomycin and 1× MITO+ (BD Biosciences, Bedford, MA, USA).

### Immunohistochemical and Periodic Acid-Schiff (PAS) Histochemical Double-staining

Formalin-fixed, paraffin-embedded tissue samples derived from melanoma patients, parallel with the primary melanoma establishment, were available at the archives of the Department of Pathology of Sheba Medical Center. Tumor tissue sections of 4 µm were prepared, warmed up to 60°C for 1 h and de-paraffinized in xylene and rehydrated. Antigen retrieval was performed using a microwave at 98°C for 16 min in 0.1 M citrate buffer pH 6. After 10 min cooling periode, the slides were rinsed in TBS buffer and an endogenous peroxidase block was performed for 10 min in 3% H_2_O_2_/MeOH. After rinses in TBS, sections were blocked with 10% goat serum for 30 min at room temperature, and incubated overnight at 4°C with the primary antibody to human CD31 (Dako, Glostrup, Denmark). Detection was performed with the Histostain-SP-Broad-Spectrum kit (Invitrogen, Grand Island, NY, USA). The antibody binding was visualized with the substrate-chromogen AEC. Then, the sections were washed with running water for 5 min and incubated with PAS (American MasterTech, CA, USA) for 15 min. All sections were counterstained with hematoxylin and cover-slipped with an aqueous mounting fluid (glycergel).

### VM and Endothelium-dependent Vessel Quantification

The CD31-PAS double-stained sections were viewed with a light microscope at a magnification of ×400 and analyzed independently by two expert pathologists. Suitable digital images were captured using brightfield digital slide scanner (3DHISTECH Ltd, Budapest, Hungary) with Panoramic Viewer software. Vessels/lined spaces stained by CD31 were defined as endothelium-dependent vessels. Channels enclosed by melanoma cells (the absence of endothelial cells confirmed by hematoxylin–eosin staining), lined by PAS-positive material and negative for CD31 immunostaining were defined as VM. The average number of VM channels and endothelium-dependent vessels in each slide was determined in areas without necrosis in 10 randomly selected fields in each slide. The proportion of VM structures out of all vessels in each of the specimens was calculated as N_VM structures_/(N_VM structures_+N_CD31-positive structures_).

### Assessment of Vasculogenic Network Formation in vitro (3D Matrix) and Image Analysis

The ability of melanoma cell lines and primary cultures to form vascular channels was assessed in vitro in three-dimensional cultures on basement membrane Matrix. Matrigel (45 µl) (BD Biosciences, Belgium) thawed on ice was dropped onto 96-well tissue culture plate and was allowed to polymerize for 45 min in a cell culture incubator [8]. 2×10^4^ tumor cells were then seeded on top of the solidified matrigel. Tube formation ability was evaluated after several hours to 24 hours and quantified by an image analysis process using whole field image capture (640 microscopic images) to avoid any bias. In principle, we quantified the distribution of the network lengths. First, a threshold was manually set to specifically demonstrate the network structures in the image. The quality and resolution of the images allowed reliable and exclusive threshold of the networks without the need of image filtering. Images were then placed in bins and subjected to the “Skeletonize” function of ImageJ software. The corresponding lengths were measured using the 2D/3D skeleton PlugIn [67] for the NIH ImageJ software [68].

### Periodic Acid-Schiff (PAS) Stain on Type I Collagen (3D Matrix)

The identification of patterned matrix type vasculogenic mimicry was performed by using type I collagen (R&D, Minneapolis, USA) following by PAS stain. 35 µl of type I collagen was dropped on 18 mm glass cover slips inserted to 12-well tissue culture plates. The collagen was allowed to polymerize for 1 hour at 37°C. 5×10^5^ cells were seeded on top of the collagen gel with overall culture medium volume of 2 ml. After seven day of cultivation a fixation was done with ethanol-formaldehyde solution for 15 min accompanied by washing with tap water for 1 min. PAS staining was carried out according to the manufacturer instruction using the material provide in the kit (Sigma-Aldrich, Israel).

### Net Cell Proliferation

Melanoma cell net proliferation was determined by standardized XTT colorimetric assay (Biological-Industries), as previously described [69]. Briefly, 3×10^6^ melanoma cells were seeded in triplicate wells in 96F-well microplates. After background subtraction, the O.D. values were transformed into viable cells counts according to the specific regression equation that was determined for each cell group examined.

### Invasion Assay

The invasive potential was quantified using matrigel-coated transwell system, as previously described [69]. Briefly, melanoma cells were harvested and re-suspended to concentration of 2×10^6^/ml in RPMI 1640 supplemented with 0.1% FBS. The cells (2×10^5^/100 µl) were then seeded into the upper wells of Transwell invasion system on Matrigel (BD Biosciences) coated ThinCerts PET 8-µm membranes (Greiner-bio-one, Germany). The lower well contained the same medium with 10% FBS. After 24 hours of incubation in humidified 5% CO2 incubator, the upper well content, which contained non-invading cells, was removed using cotton swabs. The amount of cells that invaded through the membranes was measured by standardized XTT staining (as above) and corrected for proliferation. Percent of invasion was calculated out of the number of cells seeded.

### Flow Cytometry

The expression of VE-cadherin (CD144) and VEGF-A was analyzed by flow cytometry using mouse anti-human VE-cadherin: biotin conjugate (Clone 16B1, e-Bioscience, CA, USA), FITC conjugated rabbit anti-human CD144 (SeroTec, Oxford, UK) and APC-conjugated mouse anti-human VEGF monoclonal antibody (R&D Systems, Minneapolis, MM, USA) with APC mouse IgG2b isotype control (e-Bioscience). The cells were removed with Trypsin-EDTA solution (Lonza, Verviers Sprl, Belgium), washed and re-suspended with cold PBS containing 0.5% bovine serum albumin (BSA), 2 mM EDTA and 0.002% NaN_3_ (FACS buffer) to a concentration of 2×10^6^ cells/ml. Afterward, 2×10^5^ cells were incubated on ice with the appropriate antibodies for 30 min. For VEGF-A, an intracellular staining (fixation with 2% PFA and permeabilizetion with saponine buffer −0.1% saponine; 0.2% BSA; 0.002% NaN_3_) was performed after over-night incubation with monensin (e-Bioscience, CA, USA) an intracellular protein transport blocker. Once the cells were washed (500 g, 5 min) and re-suspended with 200 µl FACS buffer, the samples were analyzed with a FACS-Calibur (Becton Dickinson, San Jose, CA) with WinMDI 2.9 Joseph Trotter Scripps data processing or with FlowJo software. The secondary antibody that was used is: PE-conjugated streptavidin (e-Bioscience).

### Proliferation Capacity and Apoptotic Cell Content

Cell cycle phase distribution according to DNA content analysis was performed using flow cytometry, by incubation of cells with propidium iodide (50 µg/ml) following the procedure of Vindelov [70]. This assay enabled to quantify the proliferative cell fraction (S+G2M) as well as the apoptotic fraction (Sub G1). The data was analyzed on FlowJO Software.

### Gene Expression Microarray Analyses

Whole genome expression oligonucleotide microarray analyses were carried out on HAG melanoma cell line treated with 20 mM nicotinamide (Sigma Aldrich) compared to vehicle-treated cells. Total RNA was extracted and processed using TRIzol® Reagent (Ambion, TX, USA), including phase separation by chloroform, RNA precipitation with isopropanol alcohol and RNA washing with 75% ethanol. The quality and integrity of the RNA were confirmed by agarose gel electrophoresis and ethidium bromide staining, followed by visual examination under ultraviolet light. Total RNA was used as template to generate cDNA with a High-capacity reverse transcriptase kit (Applied Biosystems) using random hexamer primers. Subsequent biotinylated target cRNA was processed by an Affymetrix GeneChip Instrument System (Affymetrix) according to manufacturer’s recommendations. The complete description of this procedure is available at: http://Affymetrix.com/support/technical/manual.affx. The differentially expressed genes were analyzed by Ingenuity Pathway Analysis (http://www.ingenuity.com) and Toppgene algorithm [71]. Full microarray data are deposit in NCBI GEO archive.

### Statistical Evaluation

Statistical evaluation was performed for most data by Student’s *t* test. The Likelihood ratio significance test for ordinary logistic regression was used for examines correlations between VM *in situ* (PAS positive) and VM formation *in vitro* (tube formation) and between the last and VE-cadherin expression of primary cultured melanoma cells.

## Supporting Information

Table S1 Patients clinical characteristics.
(DOC)

Table S2 Comparative whole genome expression microarray. HAG cell line treated with 20 mM NA compared to vehicle-treated cells.
(XLS)
