# Supplementary material for: Nicotinamide Inhibits Vasculogenic Mimicry, an Alternative Vascularization Pathway Observed in Highly Aggressive Melanoma
Source: PLoS One. 2013 Feb 25;8(2):e57160. doi: 10.1371/journal.pone.0057160 (PMC3581583; doi:10.1371/journal.pone.0057160)
Supplement: Table S1 — Patients clinical characteristics. (DOC) [file pone.0057160.s001.doc]

**Supplementary Table S1:** Patients clinical characteristics

| patient code | Gender | Survival stage IV  (month) | At diagnosis | | | | At time of biopsy | | | |
| --- | --- | --- | --- | --- | --- | --- | --- | --- | --- | --- |
|  |  |  | Age | stage | Breslow (mm) | Site of metastasis | Age | stage | Site of removed metastasis | Tumor size (cm^2^) |
|  |  |  |  |  |  |  |  |  |  |  |
| 001 | male | 28 | 66 | IIB | 12 | S.C. | 66 | IIB | Primary S.C. | 1.44 |
| 003 | male | 11 | 68 | IIA | unknown | LN | 68 | III | S.C., LN | 17.6 |
| 005 | female | 14 | 78 | nd | 14 | LN | 78 | IV | LN | 8.3 |
| 008 | male | nd | 56 | IIA | 3.1 | S.C. | 58 | IV | LN | 30.2 |
| 010 | female | 13 | 77 | IV | unknown | LN | 77 | IV | S.C., LN | 2.3 |
| 04 | male | 25 | 40 | II | 2.9 | S.C., LN | 49 | IV | S.C. | 47 |
| 05 | male | 10 | 57 | IIIC | 10 | LN, Lung | 57 | IV | Lung | 6.5 |
| 06 | female | 7 | 39 | IV | unknown | S.C., LN | 39 | IV | S.C., LN | 1.5 |
| 08 | Female | 29 | 33 | IV | Clear cell | LN | 34 | IV | S.C., LN | 62.7 |
| 09 | male | 40 | 53 | II | unknown | S.C., Lung | 56 | IV | S.C. | 0.9 |
| 10 | male | 10 | 31 | IIB | 8 | S.C. | 38 | IV | S.C | 0.6 |
| 12 | female | 3 | 40 | III | 3.4 | LN | 44 | IV | LN | 12.7 |
| 13 | male | 9 | 52 | IV | regression | LN, Lung | 53 | IV | S.C., LN | 3.4 |
| 14 | male | 21 | 49 | III | 4.5 | LN | 53 | IV | LN | 2.6 |
| 15 | male | nd | 51 | nd | unknown | Lung | 57 | IV | S.C. | 2.4 |

LN = Lymph node; S.C. = Subcutaneous
